# Supplementary material for: Adherence to Cervical Cancer Screening Programs in Migrant Populations: A Systematic Review and Meta-Analysis
Source: Int J Environ Res Public Health. 2023 Jan 26;20(3):2200. doi: 10.3390/ijerph20032200 (PMC9915157; doi:10.3390/ijerph20032200)
Supplement: Supplementary file 1 [file ijerph-20-02200-s001.zip › ijerph-2164087-supplementary.pdf]

Table S1: search strings for the selected databases

**PubMed**	(immigrant* OR migrant* OR foreigner* OR non-native* OR "non native" OR refugee* OR immigration) AND ("cervical cancer screening" OR "cervical screening" OR "Pap smear" OR "Pap test" OR "Pap-test" OR "papanicolaou test"[Mesh Terms]) AND (uptake OR adherence OR participation OR inclusion OR selection OR attendance OR interest)
**Scopus**	TITLE-ABS-KEY((immigrant* OR migrant* OR foreigner* OR non-native* OR "non native" OR refugee* OR immigration) AND ("cervical cancer screening" OR "cervical screening" OR "Pap smear" OR "Pap test" OR "Pap-test" OR "papanicolaou test") AND (uptake OR adherence OR participation OR inclusion OR selection OR attendance OR interest))
**Embase**	((immigrant* or migrant* or foreigner* or non-native* or "non native" or refugee* or immigration) and ("cervical cancer screening" or "cervical screening" or "Pap smear" or "Pap test" or "Pap-test" or "papanicolaou test") and (adherence or uptake or participation or inclusion or selection or attendance or interest)).mp. [mp=title, abstract, heading word, drug trade name, original title, device manufacturer, drug manufacturer, device trade name, keyword heading word, floating subheading word, candidate term word]

Table S2: characteristics of migrants and natives investigated

Author	Migrants total (n)	Screened migrants (n, %)	Adherence in migrants total (%)	Unadjusted OR for screening participation in migrants	Adjusted OR for screening participation in migrants	Adjustment	Adherence in natives (%)	OR for screening participation in natives (reference)
Aminisani et al, 2012	Asia = 8627<br>Middle East = 2850<br>*(South East Asia = 4370*<br>*North East Asia = 2903*<br>*South Central Asia = 1361)*	Asia = 5175 (60.0%)<br>Middle East = 1704 (59.8%)<br>*(South East Asia = 2705, 61.9%*<br>*North East Asia = 1774, 61.1%*<br>*South Central Asia = 696, 51.1%)*	59.9%	Asia = 0.86 (0.81-0.91)<br>Middle East = 0.85 (0.79-0.93)	Asia = 0.74 (0.70-0.79)<br>Middle East = 0.88 (0.81-0.97)	age, SES, parity, smoking, region of birth	63.50%	1.00
Azerkan et al, 2012	Northern Europe = 129143<br>Central Eastern Europe = 50549<br>Southern Europe = 60586<br>Western Europe = 22647<br>USA/Canada = 7554<br>Central America = 2475<br>South America = 22775<br>Eastern Africa = 15876<br>Middle Africa = 855<br>Northern Africa = 4978<br>Southern Africa = 619<br>Western Africa = 2654<br>Western Asia = 51690<br>South Central Asia = 34515<br>South Eastern Asia = 22768<br>Eastern Asia = 13073<br>Australia, New Zealand = 1513	Northern Europe = 69737, 54%<br>Central Eastern Europe = 26285, 52%<br>Southern Europe = 28475, 47%<br>Western Europe = 10645, 47%<br>USA/Canada = 2719, 36%<br>Central America = 1262, 51%<br>South America = 11843, 52%<br>Eastern Africa = 6192, 39%<br>Middle Africa = 419, 49%<br>Northern Africa = 2190, 44%<br>Southern Africa = 235, 38%<br>Western Africa = 1274, 48%<br>Western Asia = 24811, 48%<br>South Central Asia = 17256, 50%<br>South Eastern Asia = 10929, 48%<br>Eastern Asia = 5491, 42%<br>Australia, New Zealand = 484, 32%	49%	Northern Europe = 0.89<br>Central Eastern Europe = 0.88<br>Southern Europe = 0.79<br>Western Europe = 0.81<br>USA/Canada = 0.68<br>Central America = 0.90<br>South America = 0.90<br>Eastern Africa = 0.71<br>Middle Africa = 0.89<br>Northern Africa = 0.79<br>Southern Africa = 0.71<br>Western Africa = 0.86<br>Western Asia = 0.84<br>South Central Asia = 0.88<br>South Eastern Asia = 0.84<br>Eastern Asia = 0.79<br>Australia, New Zealand = 0.61<br>**total = 0.85**	Northern Europe = 0.92<br>Central Eastern Europe = 0.88<br>Southern Europe = 0.80<br>Western Europe = 0.86<br>USA/Canada = 0.70<br>Central America = 0.89<br>South America = 0.90<br>Eastern Africa = 0.70<br>Middle Africa = 0.89<br>Northern Africa = 0.78<br>Southern Africa = 0.73<br>Western Africa = 0.84<br>Western Asia = 0.84<br>South Central Asia = 0.88<br>South Eastern Asia = 0.83<br>Eastern Asia = 0.80<br>Australia/New Zealand = 0.63<br>**total = 0.86**	attained age, age at entry, country of residence	62%	1.00
Badre-Esfahani et al, 2020	Middle-East North Africa = 11115<br>Eastern Europe/<br>Central Asia = 3283<br>South East Asia =1923<br>Western Countries = 1028<br>Sub-Sahara = 605<br>Latin America = 151	Middle-East North Africa = 2215, 19.9%<br>Eastern Europe/<br>Central Asia = 1270, 38.7%<br>South East Asia =696, 36.2%<br>Western Countries = 470, 45.87%<br>Sub-Sahara = 248, 41%<br>Latin America = 66, 43.7%	27.2%				51.9%	
Battagello et al, 2022s	Eastern Europe = 16571<br>Asia = 5045<br>North Africa = 3036<br>Sub Saharan Africa = 1969<br>Central/Southern America = 1337	Eastern Europe = 6604, 42.4%<br>Asia = 1584, 34.6%<br>North Africa = 1061, 38%<br>Sub Saharan Africa = 760, 42.2%<br>Central/Southern America = 434, 35.7%	38.6%				49.7%	

Broberg et al, 2018	Northern Europe = 18581<br>Central/Eastern Europe = 14082<br>Southern Europe = 14103<br>Western Europe = 4369<br>North America = 1417<br>Central America/<br>Caribbean = 1008<br>South America = 5669<br>North Africa = 1913<br>East Africa = 6842<br>Central Africa = 368<br>Southern Africa = 152<br>West Africa = 985<br>West Asia = 18281<br>South Central Asia = 10414<br>South East Asia = 9275<br>East Asia = 4200<br>Australia/Melanesia = 333	Northern Europe = 8158, 43.9%<br>Central/Eastern Europe = 5189, 36.8%<br>Southern Europe = 6415, 45.5%<br>Western Europe = 1646, 37.7%<br>North America = 494, 34.9%<br>Central America/<br>Caribbean = 484, 48%<br>South America = 2631, 46.4%<br>North Africa = 673, 35.2%<br>East Africa = 1868, 27.3%<br>Central Africa = 148, 40.2%<br>Southern Africa = 51, 33.5%<br>West Africa = 411, 41.7%<br>West Asia = 7650, 41.8%<br>South Central Asia = 4193, 40.3%<br>South East Asia = 4524, 48.8%<br>East Asia = 1677, 40%<br>Australia/Melanesia = 105, 31.5%	38.7%	**OR for non participation:**<br>Northern Europe = 1.84 (1.78-1.89)<br>Central/Eastern Europe = 2.47 (2.38-2.55)<br>Southern Europe = 1.72 (1.67-1.78)<br>Western Europe = 2.38 (2.24-2.53)<br>North America = 2.69 (2.41-3.00)<br>Central America = 1.44 (1.24-1.68)<br>Caribbean = 1.82 (1.47-2.24)<br>South America = 1.66 (1.58-1.75)<br>North Africa = 2.65 (2.41-2.91)<br>East Africa = 3.81 (3.63-4.04)<br>Central Africa = 2.14 (1.74-2.64)<br>Southern Africa = 2.85 (2.04-3.99)<br>West Africa = 2.01 (1.77-2.28)<br>West Asia = 2.00 (1.94-2.06)<br>South Central Asia = 2.14 (2.05-2.22)<br>South East Asia = 1.51 (1.45-1.58)<br>East Asia = 2.17 (2.03-2.30)<br>Australia/Melanesia = 3.12 (2.48- 3.94)	**OR for non participation:**<br>Northern Europe = 1.36 (1.32-1.41)<br>Central/Eastern Europe = 1.64 (1.57-1.70)<br>Southern Europe = 1.24 (1.19-1.29)<br>Western Europe = 1.96 (1.83-2.09)<br>North America = 1.93 (1.71-2.17)<br>Central America = 0.94 (0.79-1.11)<br>Caribbean = 1.02 (0.81-1.28)<br>South America = 1.02 (0.97-1.08)<br>North Africa = 1.37 (1.24-1.52)<br>East Africa = 1.73 (1.64-1.84)<br>Central Africa = 1.08 (0.86-1.36)<br>Southern Africa = 1.99 (1.38-2.86)<br>West Africa = 0.95 (0.83-1.09)<br>West Asia = 1.01 (0.97-1.04)<br>South Central Asia = 1.30 (1.24-1.36)<br>South East Asia = 0.87 (0.83-0.91)<br>East Asia = 1.38 (1.29-1.48)<br>Australia/Melanesia = 2.13 (1.66-2.74)	age groups, county, disposable family income, labour force, unemployment benefits, welfare benefits, education, cohabiting	59%	1.00
Comparetto et al, 2017	Albania = 681<br>China = 2199	Albania = 303, 45.8%<br>China = 252, 12.5%	25.4%				60.5%	
Gallo et al, 2017	LMIC group<br>Africa = 97627<br>Asia = 32134<br>Central/Eastern Europe = 227201<br>Central/Southern America, Caribbean = 72430<br>Total LMIC group = 429392<br>HIC group (not Italy) = 70989	LMIC group<br>Africa = 40797, 41.8%<br>Asia = 11314, 35.2%<br>Central/Eastern Europe = 103642, 45.6%<br>Central/Southern America, Caribbean = 33090, 45.7%<br>Total LMIC group = 188843, 44%<br>HIC group (not Italy) = 31312, 44.1%	44%				48.6%	
Harder et al, 2018	more developed countries = 18751<br>less developed countries = 29467	more developed countries = 14372, 76.6%<br>less developed countries = 21985, 74.6%	75.6%				85.6%	
Hertzum-Larsen et al, 2019	**Western countries = 12500**<br>Western Europe = 11451<br>North America, New Zealand, Australia = 1049<br>**Non -Western countries = 44829**<br>Eastern Europe = 12892<br>Mid and Eastern Asia = 14364<br>North Africa, Western Asia = 12749<br>South and Central America = 1396<br>Sub-Saharan Africa = 3429	**Western countries = 7537, 61.2%**<br>Western Europe = 7078, 62.8%<br>North America, New Zealand, Australia = 459, 44.3%<br>**Non -Western countries = 27307, 61.3%**<br>Eastern Europe = 7831, 61.3%<br>Mid and Eastern Asia = 8773, 61.5%<br>North Africa, Western Asia = 8042, 63.3%<br>South and Central America = 938, 67.8%<br>Sub-Saharan Africa = 1723, 50.6%	61%	**HR reported**<br>**Western countries = 0.73 (0.71-0.74)**<br>Western Europe = 0.75 (0.73-0.77)<br>North America, New Zealand, Australia = 0.48 (0.44-0.53)<br>**Non -Western countries = 0.70 (0.70-0.71)**<br>Eastern Europe = 0.71 (0.70-0.73)<br>Mid and Eastern Asia = 0.72 (0.70-0.73)<br>North Africa, Western Asia = 0.72 (0.71-0.74)<br>South and Central America = 0.84 (0.79-0.90)<br>Sub-Saharan Africa = 0.52 (0.50-0.55)	**HR reported**<br>**Western countries = 0.79 (0.77-0.81)**<br>Western Europe = 0.81 (0.79-0.83)<br>North America, New Zealand, Australia = 0.55 (0.51-0.61)<br>**Non -Western countries = 0.77 (0.76-0.78)**<br>Eastern Europe = 0.76 (0.75-0.78)<br>Mid and Eastern Asia = 0.77 (0.76-0.79)<br>North Africa, Western Asia = 0.79 (0.78-0.81)<br>South and Central America = 0.90 (0.85-0.96)<br>Sub-Saharan Africa = 0.61 (0.58-0.64)	age, employment, income, civil status, schizophrenia, alcohol, intoxicant abuse, affective disorders, anxiety, adjustment disorders, parity, Charlson comorbidity index	74.5%	1.00
Idehen et al, 2020	Russian = 816<br>Somali = 523<br>Kurdish = 451	Russian = 516, 63.3%<br>Somali = 99, 18.9%<br>Kurdish = 311, 68.9%	50.40%		Russian = 0.92 (0.74-1.16)<br>Somali = 0.16 (0.11-0.22)<br>Kurdish = 1.37 (1.02-1.83)	study group, age, education, living abroad, hospital care, employment status	66.6%	1.00

Leinonen et al, 2017	**Nordic countries = 23243**<br>Sweden = 12022<br>Denmark = 5831<br>Finland = 2959<br>**Eastern Europe = 60219**<br>Poland = 19206<br>Russia = 7762<br>Lithuania = 7645<br>Bosnia-Herzegovina = 5050<br>Kosovo = 3192<br>Romania = 3012<br>**Western Europe = 18381**<br>Germany = 7650<br>UK = 3254<br>the Netherlands = 2124<br>**America, Oceania = 12845**<br>USA = 2707<br>Chile = 2563<br>Brasil = 2142<br>**Western Asia, North Africa = 16361**<br>Iraq = 5932<br>Turkey = 4222<br>Morocco = 2165<br>**Sub-saharan Africa = 17584**<br>Somalia = 6414<br>Eritrea = 3344<br>**South Central Asia = 26719**<br>Pakistan = 8957<br>Iran = 5528<br>India = 3489<br>SriLanka = 3692<br>Afganistan = 2528<br>**Eastern Asia = 33274**<br>Philippines = 10565<br>Thailand = 10465<br>Vietnam = 6485<br>China = 3257	**Nordic countries = 61.1%**<br>Sweden = 62.5%<br>Denmark = 63%<br>Finland = 59.4%<br>**Eastern Europe = 38.6%**<br>Poland = 30.4%<br>Russia = 53.4%<br>Lithuania = 21.9%<br>Bosnia-Herzegovina = 56.7%<br>Kosovo = 57.1%<br>Romania = 36.7%<br>**Western Europe = 51.8%**<br>Germany = 50.3%<br>UK = 57.4%<br>the Netherlands = 59.9%<br>**America, Oceania = 57.3%**<br>USA = 57.4%<br>Chile = 58.6%<br>Brasil = 59.2%<br>**Western Asia, North Africa = 54.9%**<br>Iraq = 59.8%<br>Turkey = 55.8%<br>Morocco = 52.7%<br>**Sub-saharan Africa = 46.5%**<br>Somalia = 41.5%<br>Eritrea = 40.7%<br>**South Central Asia = 49.5%**<br>Pakistan = 46.3%<br>Iran = 58.4%<br>India = 46.7%<br>SriLanka = 48.7%<br>Afganistan = 51%<br>**Eastern Asia = 44.9%**<br>Philippines = 38.4%<br>Thailand = 44.9%<br>Vietnam = 55.1%<br>China = 45.9%	51%				68%	
Lofters et al, 2015	7737	5370, 69.4%	69.40%				71.90%	
Pankakoski et al, 2020	Swedish = 57476<br>Russian = 22352<br>Estonian = 11079<br>Thai = 4144<br>Chinese = 2663<br>English = 2478<br>Somali = 2049<br>other = 26808	Swedish = 39199, 68.2%<br>Russian = 14529, 65.0%<br>Estonian = 6027, 54.4%<br>Thai = 2466, 59.5%<br>Chinese = 1334, 50.1%<br>English = 1080, 43.6%<br>Somali = 512, 25%<br>other = 13511, 50.4%	52.02%				69.4%	
Rodvall et al, 2005	The Nordic countries (no Sweden) = 22742<br>EU 15 (no Denmark, Finland, Sweden) = 6158<br>Europe (no EU15 and The Nordic countries) = 9873<br>Africa = 4410<br>North America = 1702<br>Latin and South America = 4728	The Nordic countries (no Sweden) = 13691, 60.2%<br>EU 15 (no Denmark, Finland, Sweden) = 3024, 49.1%<br>Europe (no EU15 and The Nordic countries) = 5499, 55.7%<br>Africa = 2187, 49.6%<br>North America = 691, 40.6%<br>Latin and South America = 2685, 56.8%	51.3%				55.37%	

	Asia = 16848<br>Oceania = 207<br>The Former Soviet Union = 908	Asia = 9704, 57.6%<br>Oceania = 74, 35.7%<br>The Former Soviet Union = 516, 58.6%						
Virtanen et al, 2015	other = 1818	other = 1283, 70.6%	70.60%		0.89 (0.84-0.95)	age, mother tongue, municipality, education level, marital status, geographic location	80.35%	1.00
Visioli et al, 2015	**countries of advanced development = 14300**<br>European Union = 8992<br>Other European countries = 1910<br>Asia = 1056<br>North America = 2047<br>Oceania = 295<br>**countries of high migration pressure = 37981**<br>Central/Eastern Europe = 14307<br>South/Central America = 7597<br>West Asia (no Israel) = 745<br>Central/South Asia = 1954<br>East Asia (no Japan) = 7235<br>North Africa = 2949<br>West Africa = 1087<br>East Africa = 1864<br>Central/South Africa = 248	**countries of advanced development = 6001, 41.3%**<br>European Union = 3812, 42%<br>Other European countries = 822, 42.2%<br>Asia = 467, 41.7%<br>North America = 774, 32.1%<br>Oceania = 126, 41.9%<br>**countries of high migration pressure = 14093, 34%**<br>Central/Eastern Europe = 6204, 43.4%<br>South/Central America = 3324, 43.8%<br>West Asia (no Israel) = 239, 32.1%<br>Central/South Asia = 535, 27.4%<br>East Asia (no Japan) = 1641, 22.7%<br>North Africa = 1145, 38.8%<br>West Africa = 390, 35.8%<br>East Africa = 531, 28.5%<br>Central/South Africa = 84, 33.9%	37.7%		countries of advanced development = 0.89 (0.87-0.91)<br>countries of high migration pressure = 0.80 (0.79-0.81)	age class	46.7%	1.00
Webb et al, 2004	Caribbean = 436<br>South Asia = 2236<br>Ireland = 1078<br>Africa "other" = 1083<br>North America, New Zealand, Australia = 279<br>Europe "other" = 609<br>Central/South America = 146<br>Somalia = 243<br>China = 671<br>Middle East/Arab Gulf = 666<br>Eastern Europe/Russia = 187<br>Greece/Turkey/Cyprus = 150<br>South East Asia = 533<br>North Africa = 400<br>Asia "other" = 196	Caribbean = 287, 65.8%<br>South Asia = 1465, 65.5%<br>Ireland = 666, 61.8%<br>Africa "other" = 651, 60.1%<br>North America, New Zealand, Australia = 159, 57%<br>Europe "other" = 347, 57%<br>Central/South America = 77, 52.7%<br>Somalia = 128, 52.7%<br>China = 349, 52%<br>Middle East/Arab Gulf = 338, 50.8%<br>Eastern Europe/Russia = 94, 50.3%<br>Greece/Turkey/Cyprus = 71, 47.3%<br>South East Asia = 240, 45%<br>North Africa = 175, 53.8%<br>Asia "other" = 73, 37.2%	57.40%				75.6%	
Yeasmeen et al, 2019	Muslim countries = 77876<br>non-Muslim countries = 422091	Muslim countries = 22023, 34.7%<br>non-Muslim countries = 149842, 35.5%	35.1%	Muslim countries = 0.77 (0.75-0.78)<br>non-Muslim countries = 0.83 (0.82-0.84)			37.7%	1.00

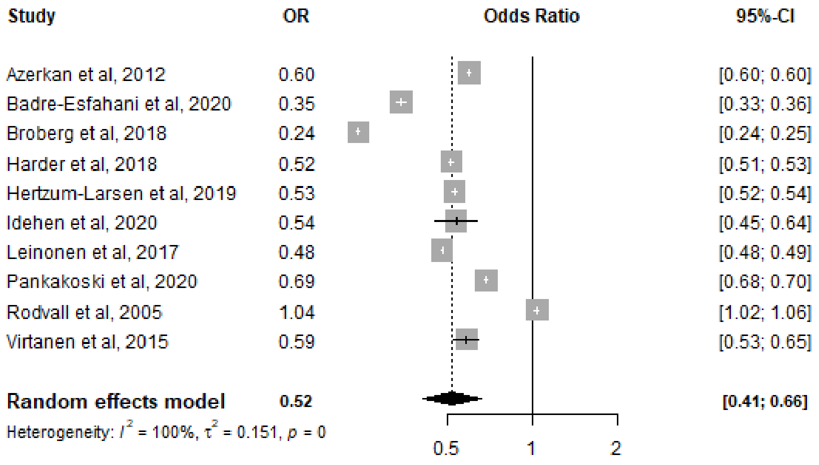

Figure S1. forest plot presenting studies conducted in North Europe only (Sweden, Norway, Finland, Denmark) [33,34,36,39–45].

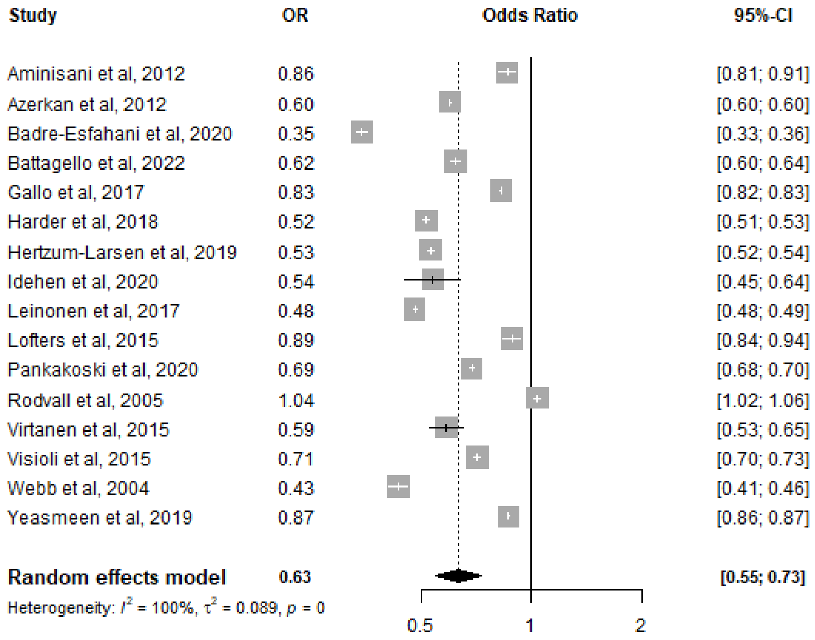

Figure S2: forest plot with the exclusion of potential outliers according to examination of studentized residuals [16,25,33–35,38–48]

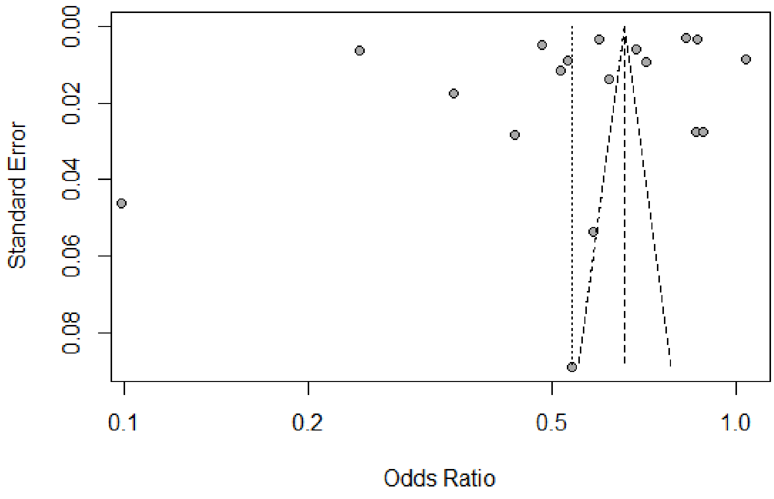

Figure S3: funnel plot for included studies

Table S3: PRISMA checklist

PRISMA 2020 Main Checklist

Topic	No.	Item	Location where item is reported
TITLE			
Title	1	Identify the report as a systematic review.	page 1
ABSTRACT			
Abstract	2	See the PRISMA 2020 for Abstracts checklist	Abstract checklist
INTRODUCTION			
Rationale	3	Describe the rationale for the review in the context of existing knowledge.	page 1-2
Objectives	4	Provide an explicit statement of the objective(s) or question(s) the review addresses.	page 2
METHODS			
Eligibility criteria	5	Specify the inclusion and exclusion criteria for the review and how studies were grouped for the syntheses.	page 2-3
Information sources	6	Specify all databases, registers, websites, organizations, reference lists and other sources searched or consulted to identify studies. Specify the date when each source was last searched or consulted.	page 2
Search strategy	7	Present the full search strategies for all databases, registers and websites, including any filters and limits used.	Table S1
Selection process	8	Specify the methods used to decide whether a study met the inclusion criteria of the review, including how many reviewers screened each record and each report retrieved, whether they worked independently, and if applicable, details of automation tools used in the process.	page 3
Data collection process	9	Specify the methods used to collect data from reports, including how many reviewers collected data from each report, whether they worked independently, any processes for obtaining or confirming data from study investigators, and if applicable, details of automation tools used in the process.	page 3-4
Data items	10a	List and define all outcomes for which data were sought. Specify whether all results that were compatible with each outcome domain in each study were sought (e.g. for all measures, time points, analyses), and if not, the methods used to decide which results to collect.	page 4
	10b	List and define all other variables for which data were sought (e.g. participant and intervention characteristics, funding sources). Describe any assumptions made about any missing or unclear information.	page 4
Study risk of bias assessment	11	Specify the methods used to assess risk of bias in the included studies, including details of the tool(s) used, how many reviewers assessed each study and whether they worked independently, and if applicable, details of automation tools used in the process.	n/a
Effect measures	12	Specify for each outcome the effect measure(s) (e.g. risk ratio, mean difference) used in the synthesis or presentation of results.	page 4
Synthesis methods	13a	Describe the processes used to decide which studies were eligible for each synthesis (e.g. tabulating the study intervention characteristics and comparing against the planned groups for each synthesis (item 5)).	page 4
	13b	Describe any methods required to prepare the data for presentation or synthesis, such as handling of missing summary statistics, or data conversions.	page 4
	13c	Describe any methods used to tabulate or visually display results of individual studies and syntheses.	page 4

Topic	No.	Item	Location where item is reported
	13d	Describe any methods used to synthesize results and provide a rationale for the choice(s). If meta-analysis was performed, describe the model(s), method(s) to identify the presence and extent of statistical heterogeneity, and software package(s) used.	page 4
	13e	Describe any methods used to explore possible causes of heterogeneity among study results (e.g. subgroup analysis, meta-regression).	page 4
	13f	Describe any sensitivity analyses conducted to assess robustness of the synthesized results.	page 4
Reporting bias assessment	14	Describe any methods used to assess risk of bias due to missing results in a synthesis (arising from reporting biases).	n/a
Certainty assessment	15	Describe any methods used to assess certainty (or confidence) in the body of evidence for an outcome.	n/a
RESULTS			
Study selection	16a	Describe the results of the search and selection process, from the number of records identified in the search to the number of studies included in the review, ideally using a flow diagram.	page 4-5
	16b	Cite studies that might appear to meet the inclusion criteria, but which were excluded, and explain why they were excluded.	page 5
Study characteristics	17	Cite each included study and present its characteristics.	page 6-7-8, Table2, Table S2
Risk of bias in studies	18	Present assessments of risk of bias for each included study.	n/a
Results of individual studies	19	For all outcomes, present, for each study: (a) summary statistics for each group (where appropriate) and (b) an effect estimate and its precision (e.g. confidence/credible interval), ideally using structured tables or plots.	page 9, Table S2
Results of syntheses	20a	For each synthesis, briefly summarize the characteristics and risk of bias among contributing studies.	page 9-10
	20b	Present results of all statistical syntheses conducted. If meta-analysis was done, present for each the summary estimate and its precision (e.g. confidence/credible interval) and measures of statistical heterogeneity. If comparing groups, describe the direction of the effect.	page 9-10
	20c	Present results of all investigations of possible causes of heterogeneity among study results.	page 10, Figure S1,S2
	20d	Present results of all sensitivity analyses conducted to assess the robustness of the synthesized results.	page 10, Figure S1, S2
Reporting biases	21	Present assessments of risk of bias due to missing results (arising from reporting biases) for each synthesis assessed.	n/a
Certainty of evidence	22	Present assessments of certainty (or confidence) in the body of evidence for each outcome assessed.	n/a
DISCUSSION			
Discussion	23a	Provide a general interpretation of the results in the context of other evidence.	page 11-12
	23b	Discuss any limitations of the evidence included in the review.	page 12
	23c	Discuss any limitations of the review processes used.	page 12
	23d	Discuss implications of the results for practice, policy, and future research.	page 12
OTHER INFORMATION			
Registration and protocol	24a	Provide registration information for the review, including register name and registration number, or state that the review was not registered.	n/a
	24b	Indicate where the review protocol can be accessed, or state that a protocol was not prepared.	n/a
	24c	Describe and explain any amendments to information provided at registration or in the protocol.	n/a
Support	25	Describe sources of financial or non-financial support for the review, and the role of the funders or sponsors in the review.	page 13
Competing interests	26	Declare any competing interests of review authors.	page 13
Availability of data, code and other materials	27	Report which of the following are publicly available and where they can be found: template data collection forms; data extracted from included studies; data used for all analyses; analytic code; any other materials used in the review.	page 13

PRISMA Abstract Checklist

Topic	No.	Item	Reported?
TITLE			
Title	1	Identify the report as a systematic review.	Yes
BACKGROUND			
Objectives	2	Provide an explicit statement of the main objective(s) or question(s) the review addresses.	Yes
METHODS			
Eligibility criteria	3	Specify the inclusion and exclusion criteria for the review.	Yes
Information sources	4	Specify the information sources (e.g. databases, registers) used to identify studies and the date when each was last searched.	Yes
Risk of bias	5	Specify the methods used to assess risk of bias in the included studies.	No
Synthesis of results	6	Specify the methods used to present and synthesize results.	Yes

Topic	No.	Item	Reported?
RESULTS			
Included studies	7	Give the total number of included studies and participants and summarize relevant characteristics of studies.	Yes
Synthesis of results	8	Present results for main outcomes, preferably indicating the number of included studies and participants for each. If meta-analysis was done, report the summary estimate and confidence/credible interval. If comparing groups, indicate the direction of the effect (i.e. which group is favored).	Yes
DISCUSSION			
Limitations of evidence	9	Provide a brief summary of the limitations of the evidence included in the review (e.g. study risk of bias, inconsistency and imprecision).	Yes
Interpretation	10	Provide a general interpretation of the results and important implications.	Yes
OTHER			
Funding	11	Specify the primary source of funding for the review.	No
Registration	12	Provide the register name and registration number.	No
